# Non-surgical management of vesicoureteral junction obstruction: a case report

**DOI:** 10.1590/2175-8239-JBN-2020-0152

**Published:** 2021-02-12

**Authors:** Thais Yuki Kimura, Pedro Alves Soares Vaz de Castro, Thiago Vasconcelos Silva, Jordana Almeida Mesquita, Ana Cristina Simões e Silva

**Affiliations:** 1 Universidade Federal de Minas Gerais, Faculdade de Medicina, Departamento de Pediatria, Unidade de Nefrologia Pediátrica, Belo Horizonte, MG, Brasil.

**Keywords:** Hydronephrosis, Kidney Function Tests, Radionuclide Imaging, Hidronefrose, Testes de Função Renal, Cintilografia

## Abstract

**Objective:**

To report the case of a pediatric patient with bilateral hydronephrosis due to vesicoureteral junction obstruction (VUJO) that was treated non-surgically and to discuss the approach of this anomaly.

**Case Description:**

A 25-month-old boy was referred without complaints for consultation due to prenatal ultrasound showing kidneys with cysts. He was under antibiotic prophylaxis. No family history of kidney disease and/or inherited disorders was reported. Renal ultrasound (RUS) at 2 days of life showed bilateral hydronephrosis, thus ruling out the possibility of kidney cystic disease. Dynamic renal scintigraphy (DTPA) showed marked retention of the marker in the pyelocaliceal system bilaterally, with little response to diuretic drug. He was maintained under antibiotic prophylaxis, when a new RUS showed bilateral ureteral dilatation, abrupt stenosis in the ureterovesical transition region (0.2 cm caliber), moderate bilateral hydronephrosis, and slight renal cortical thickness, confirming the diagnosis of VUJO. At 2 years and 10 months of age, DTPA showed hydronephrosis and ureteral stasis in both kidneys secondary to stenosis at the vesicoureteral junction (VUJ) level, with preservation of kidney function and slow degree of emptying. We opted for a non-surgical approach. RUS at 10 years of age showed significant improvement of all parameters, with ureteral transverse diameter of 9 mm, preserved VUJ, and age-appropriate bilateral kidney development.

**Comments:**

VUJO is a major cause of prenatal hydronephrosis and can trigger a deterioration of kidney function. Its treatment is still controversial but should take into account the importance of clinical follow-up and serial imaging evaluation.

## Resumo

**Objetivo:**

Relatar o caso de um paciente pediátrico com hidronefrose bilateral devido à obstrução da junção vesicoureteral (OJVU) que foi tratado não cirurgicamente e discutir a abordagem desta anomalia.

**Descrição do caso:**

Um menino de 25 meses de idade foi encaminhado sem queixas para consulta devido à ultrassonografia pré-natal que mostrou rins com cistos. Ele estava sob profilaxia antibiótica. Nenhum histórico familiar de doença renal e/ou distúrbios hereditários foi relatado. A ultrassonografia renal (USR) aos 2 dias de vida mostrou hidronefrose bilateral, descartando assim a possibilidade de doença renal cística. A cintilografia renal dinâmica (DTPA) mostrou uma retenção acentuada do marcador no sistema pielocalicial bilateralmente, com pouca resposta ao diurético. O paciente foi mantido em profilaxia antibiótica, quando uma nova USR apresentou dilatação ureteral bilateral, estenose abrupta na região de transição ureterovesical (calibre 0,2 cm), hidronefrose bilateral moderada e leve espessura cortical renal, confirmando o diagnóstico de OJVU. Aos 2 anos e 10 meses de idade, a DTPA mostrou hidronefrose e estase ureteral em ambos os rins secundárias à estenose no nível da junção vesicoureteral (JVU), com preservação da função renal e grau lento de esvaziamento. Optamos por uma abordagem não cirúrgica. A USR aos 10 anos de idade mostrou melhora significativa de todos os parâmetros, com diâmetro transverso ureteral de 9 mm, JVU preservada, e desenvolvimento renal bilateral adequado à idade.

**Comentários:**

A OJVU é uma das principais causas de hidronefrose pré-natal e pode desencadear uma deterioração da função renal. Seu tratamento ainda é controverso, mas deve levar em consideração a importância do acompanhamento clínico e da avaliação seriada por imagem.

## Introduction

With an estimated incidence of 36 cases per 100,000 births and higher prevalence in the male sex^1^, vesicoureteral junction obstruction (VUJO) is characterized by a total or partial obstruction of urine flow in the distal portion of the ureters. VUJO is one of the conditions included in the heterogeneous group of congenital anomalies of the kidney and urinary tract (CAKUT)^2^. Although still uncertain, its pathogenesis is associated with an abnormality or delay in the development of the muscles of the distal ureteral portion during the 20th week of pregnancy^3^. It is considered the second leading cause of prenatal hydronephrosis^2^, defined as the dilation of the renal calyces and/or pelvis, which can lead to a progressive deterioration of kidney function and, consequently, irreversible damage to the organ.

With the advent of modern imaging technologies and the widespread use of prenatal ultrasound, the diagnosis of CAKUT has been done earlier and more often^4^^,^ 5. The treatment, however, has undergone few changes over time and, on some occasions, it still involves surgical intervention^6^, with cutaneous ureterostomy^7^, ureteral reimplantation^8^, and nephrectomy as the most widely used techniques in cases of loss of kidney function^6^. Furthermore, new and less invasive endoscopic techniques emerged as novel surgical approaches^6^^,^ 9.

Given the uncertainty of the most appropriate treatment for this congenital anomaly and due to its potential impact on the patient's quality of life, our objective was to report the case of a pediatric patient with bilateral hydronephrosis due to vesicoureteral junction obstruction (VUJO) that was treated non-surgically and to discuss the literature related to the approach of this anomaly.

## Methods

This study is a case report in accordance with the Helsinki Declaration and a literature review. The patient's guardians signed a consent form authorizing the case report for scientific purpose. The case information was obtained through the review of the patient's medical records and imaging exams. For the literature review, PubMed (MEDLINE), LILACS, and SciELO databases were searched, using the descriptors "vesicoureteral junction" and "pediatrics", both listed on the Medical Headings Subjects (MeSH). Other studies related to the aforementioned search were also used.

## Case Description

**Figure 1:**
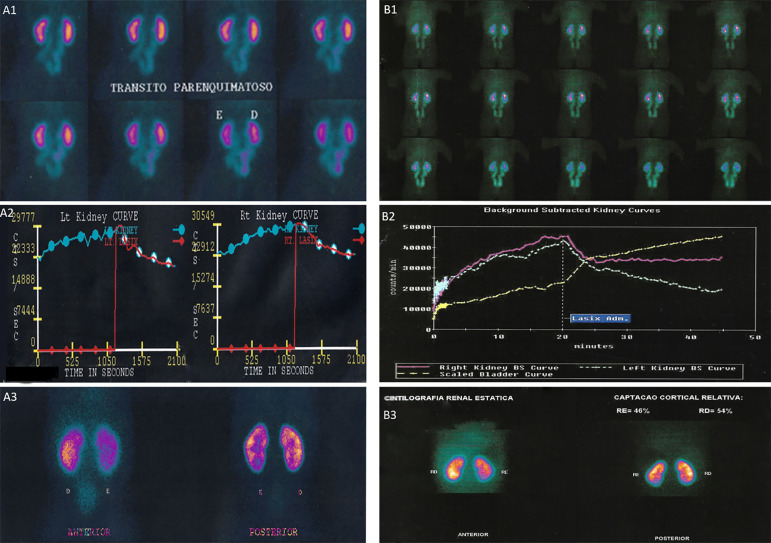
Dynamic and static renal scintigraphy at 5 months of age (A1, A2, A3) and 3 years of age **(B1, B2, B3)** showing scintigraphic pattern compatible with bilateral hydronephrosis secondary to stasis at the level of the vesicoureteral junction (VUJ) with preservation of kidney function. **A1, B1.** Dynamic renal scintigraphy showing retention of the radiotracer in the pyelocaliceal system bilaterally (rate of 1 minute/frame). **A2, B2.** Dynamic renal scintigraphy curves also showing retention of the DTPA marker, even with administration of furosemide, and incomplete drainage of the excretory system, due to partial obstruction of VUJ, considering the slow descending pattern of the renogram curve. **A3, B3.** Static renal scintigraphy showing preserved kidney function and symmetrical radiotracer expression with relative kidney function, being left kidney of 46% and right kidney of 54%.

A 25-month-old boy was referred to our outpatient clinic without complaints due to a gestational ultrasound that showed kidney cysts. The patient was already under antibiotic prophylaxis and physical examination and vital signs within normal limits. The parents reported no family history of kidney disease and/or other heredofamilial disorders. Renal ultrasound performed at 2 days of life showed bilateral hydronephrosis, mainly on the right kidney. At 5 months of age, a DTPA and a DMSA were performed. The DTPA showed strong retention of the marker in the pyelocalyceal system bilaterally, with little response to diuretic stimulus (Figure 1. A1, A2), while the DMSA indicated preserved kidney function and symmetrical radiotracer distribution in kidneys with relative kidney function of 46% in the left kidney and 54% in the right kidney (Figure 1. A3).

**Figure 2:**
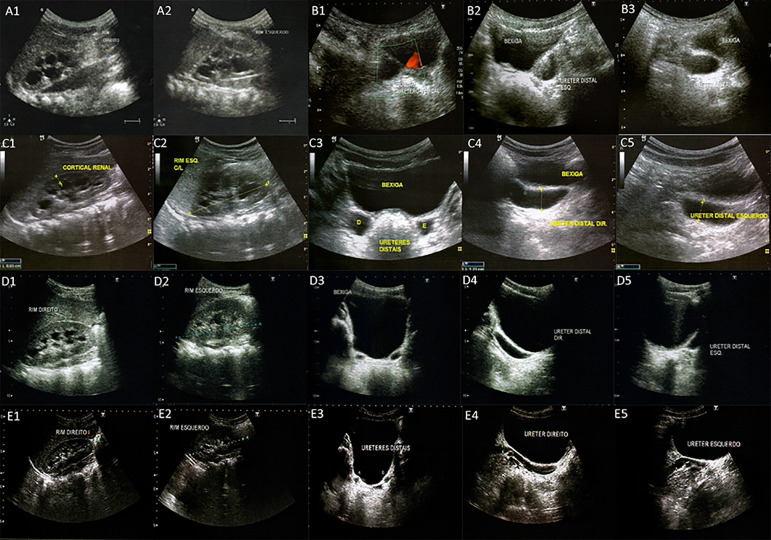
Renal ultrasound showing patient evolution according to serial ultrasonography (US) evaluation. **A1, A2.** US at 1-month of age showing bilateral hydronephrosis, mainly on the right kidney. Right kidney size: W 50mm x L 29mm x T 20mm. Left kidney size: W 57mm x L 26mm x T 27mm. **B1, B2, B3.** At 1 year of age, moderate bilateral hydronephrosis with slight reduction of renal cortex thickness and dilation of both ureters entire length, except at the ureteral vesical junction (UVJ), which exhibits a constriction area. Right kidney size: W 65mm x L 34mm x T 33mm. Left kidney size: W 69mm x L 32mm x T 29mm. **C1, C2, C3, C4, C5.** At 2 years of age, moderate bilateral hydronephrosis, still with slight thinning of renal cortex and dilation of both ureters entire lenght (1 cm on the right side and 0.8 cm on the left), with an abrupt stenosis in the UVJ region of 0.2 cm caliber. Right kidney size: 76mm x 28mm x 28mm. Left kidney size: W 69mm x L 30mm x T 30mm. **D1, D2, D3, D4, D5.** At 7 years of age, moderate bilateral dilation of both ureters with diameter of 0.8cm and still with important reduction of the distal ureters and UVJ caliber. Mild dilation of the pyelocaliceal system. Renal dimensions within normal values. Right kidney size: W 81mm x L 32mm x T 32mm. Left kidney size: W 82mm x L 34mm x T 33mm. **E1, E2, E3, E4, E5.** At 12 years, mild pyelocaliceal system ectasia and reduced ureteral dilation. Both ureters with preserved trajectory and with estimated diameter of 0.7cm. Preserved renal cortex.

Based on the first appointment at our clinic, the antibiotic prophylaxis was maintained and a new kidney ultrasound was requested, which showed dilation in the entire length of both ureters (10 mm on the right side and 8 mm on the left), with an abrupt stenosis in the distal region of both ureters at the bladder entrance with a caliber of only 2 mm. In addition, moderate bilateral hydronephrosis and slight thinning of the renal cortex parenchyma were also observed (Figure 2. C2-C5). These findings indicated the diagnosis of bilateral megaureter secondary to stenosis caused by VUJO and vesicoureteral reflux (VUR). At 2 years and 10 months of age, DTPA and DMSA were requested. The DTPA showed obstructive pyelocalyceal and urethral stasis in both kidneys, with a scintigraphic pattern compatible with bilateral hydronephrosis secondary to stasis at the level of the VUJ with preservation of kidney function (Figure 1. B1, B2). Despite the observed dilation, the parenchyma and renal cortex were preserved and the pattern of DTPA showed a slow emptying degree, while the DMSA showed that relative kidney function remained stable at 46:54 (Figure 1. B3).

Taking into account the clinical and imaging findings, non-surgical management was adopted, with clinical follow-up and serial imaging evaluation. During the first year, the patient visited the ambulatory semiannually and in the following 2 years, annually. At 7 years and 7 months, antibiotic prophylaxis was discontinued, with no history of urinary tract infections, and follow-up visits were maintained every 2 years. Kidney ultrasonography at 10 years of age showed significant improvement of all parameters with ureteral transverse diameter exhibiting a slight to moderate increase (0.9 cm) and preserved VUJ, indicating a satisfactory evolution and expected bilateral kidney development with the non-surgical approach. For the entire follow-up time, the patient stayed normotensive, with normal serum urea and creatinine levels, and without proteinuria, indicating favorable clinical evolution.

## Discussion

In this study, we reported a case of bilateral hydronephrosis detected at prenatal ultrasound and diagnosed as VUJO at 2 years of age. The literature has showed a tendency for early diagnosis of this anomaly due to the development of ultrasound techniques^10^. In addition, the use of renal scintigraphy exams was important for the follow-up of this case, since this technique allowed the evaluation of patient's kidney function, which is considered essential to verify the effects of obstructive megaureter in renal parenchyma. It should be mentioned that, in general, obstructive uropathies are responsible for approximately 27% of the causes of end stage renal disease in pediatric patients, with 3.5% of these cases caused by VUJO^11^.

Most cases of hydronephrosis due to VUJO associated with megaureter without VUR may present spontaneous resolution^12^. However, there is no consensus on the criteria for surgical intervention. Cox proportional hazards regression has been applied to assess the association between candidate predictors, such as eGFR, associated hydronephrosis, kidney damage, and the severity of dilatation of renal pelvis, and the need of surgery in children with prenatally detected CAKUT^13^. In addition, some possible indications for surgery, according to the classification of the Society for Fetal Urology (SFU) of 2015^14^, include grade 4 or 5 of hydronephrosis, in which there is dilation of the renal calyces with impaired renal parenchyma^12^. The measurement of the anteroposterior diameter of the renal pelvis (APD), with or in combination with diffuse caliectasis, has also been proposed as a predictor for surgical intervention, mainly in cases with moderate APD values (≥6-9 mm and ≤9-15 mm)^14^.

Although the reported case presented a large dilation of the ureter diameter (10 mm), which can be considered for surgical indication^12^, priority was given for a non-invasive patient management, since non-surgical treatment is currently preferred in about 85 to 90% of megaureter cases due to primary obstruction^15^. Furthermore, the patient's normotension and absence of proteinuria during the follow-up indicated low probability of chronic kidney disease (CKD) development^16^.

## Conclusion

Despite being considered safe and presenting a good prognosis, the surgical procedure for hydronephrosis in VUJO is still an invasive method that in many cases can be dispensable. In the present case, the relevance of clinical follow-up and serial imaging evaluation of the disease's evolution was proved efficient for an adequate and less invasive approach, preserving the patient's renal function.
